# Editorial: Advances in Applied Bioinformatics in Crops

**DOI:** 10.3389/fpls.2021.640394

**Published:** 2021-02-12

**Authors:** Mary-Ann Blätke, Jedrzej Jakub Szymanski, Evgeny Gladilin, Uwe Scholz, Sebastian Beier

**Affiliations:** ^1^Department of Molecular Genetics, Leibniz Institute of Plant Genetics and Crop Plant Research (IPK), Seeland, Germany; ^2^Department of Breeding Research, Leibniz Institute of Plant Genetics and Crop Plant Research (IPK), Seeland, Germany

**Keywords:** breeding informatics, image analysis, data visualization, biodiversity, plant (multi)omics, computational biology, high-throughput technologies in plants

## Introduction

Big Data in life science is scattered across hundreds of unstructured data sets, biological databases and thousands of scientific journals. Modern crop research relies on high-throughput technologies that generate large quantities of high-dimensional data. The challenge for Applied Bioinformatics is to capture, model, integrate, analyze, visualize and make these data accessible in a FAIR (Wilkinson et al., 2016) (https://fair-dom.org) way. This, in turn, translates directly to the improvement of our understanding of crop biology, and in practical terms results in the development of new elite genotypes and improvement of plant cultivation strategies.

The presented collection of articles describes the flow of information from high-throughput data acquisition, data processing and analysis, the underlying IT infrastructure, and the modeling of biological processes. This Research Topic (RT) special issue is based on contributions to the Fifteenth *Gatersleben Research Conference on Applied Bioinformatics in Crops* carried out during March 2019 at the Leibniz Institute of Plant Genetics and Crop Plant Research (IPK) in Gatersleben, Germany, and includes contributions from scientists who are researching related topics. This conference was of interest to Life Scientists, Bioinformaticians, Computer Scientists, Systems Biologists, Synthetic Biologists, and others working or interested in the developing area of Applied Bioinformatics for crops.

Advances in high-throughput technologies, such as next-generation sequencing (NGS) have paved the way for turning life sciences into data-intensive disciplines. NGS has enabled leaps forward in plant genomics by tremendously increasing the number of sequenced genomes and assembling pan genomes to explore genomic diversity (Bayer et al., 2020). Other disciplines such as genome-wide association studies (GWAS), transcriptomic, proteomic, and metabolomic profiling benefit from advanced high-throughput experimental techniques and other omic-related fields. Those advances would not have been possible without bioinformatics providing novel tools, databases, and the other resources required to analyze the ever-increasing amounts of data. Nevertheless, extracting the inherent valuable knowledge hidden within numerous, large and diverse data sets remains a daunting challenge in bioinformatics and computational biology (Tang et al., 2019). In this respect, deep learning advances the bioinformatics toolkit and is of unprecedented value to unveil precious insights linked to genetic information, molecules and molecular processes. Deep learning in plant sciences allows us to conduct high-throughput phenotyping based on classical image data, as well as complex comparative genomic, transcriptomic, and proteomic studies, see Soltis et al. (2020) and Tang et al. (2019) for a review. The systematic management of scientific and research data, long-term data storage, backup and accessibility, allowing us to network data both nationally, and internationally is increasingly important. The bioinformatics community pushes forward implementing FAIR principles and open science into practice, such as the plant phenomics and genomics repository (Arend et al., 2016).

The various topics discussed during this RT can be summarized into four major categories. (1) **Biodiversity and information systems** includes contributions regarding diversity studies and the embedding of data in information systems. (2) **Distributed computing, tools and infrastructures**, on the other hand, addresses the description, benchmarking and fundamental IT infrastructure of new research software tools and pipelines. (3) **Breeding informatics** is about insights into genome sequence analysis and new challenges in plant breeding. (4) **Image-based analysis and data visualization** presents methods and tools that can be used in the optical and exploratory analysis of plant traits.

## Contributions

The study by Leidenfrost et al. investigates bumble bees as important crop pollinators, examining their food preferences by collecting and sequencing pollen samples. They also compared the results of Illumina short-read technology with Nanopore MinION sequencing. Due to the error-prone nature of Nanopore data, interpretation of these results was more challenging than those of Illumina data. However, the authors were able to conclude that there were fewer errors from the short-read sequencing data, which enabled the discovery of shorter genetic markers in Illumina data in contrast to Nanopore data. This revealed that bumble bees require greater plant diversity than only crops to meet their foraging preferences [relating to the sub-categories of this RT on (1) Biodiversity and information systems and (2) Distributed computing, tools and infrastructures].

Different Illumina marker platforms were used by Soleimani et al. to analyze the effects of core set selection methods in wheat. For this purpose, they introduced a new 15K SNP array, focussing on providing a reliable and cost-effective alternative to other available platforms. They were able to show that the popular *k*-medoids method performs as well as other core selection methods, such as Core Hunter 3 (De Beukelaer et al., 2018) to capture the diversity of a population in a smaller core set [categories (1) Biodiversity and information systems, (2) Distributed computing, tools and infrastructures, and (3) Breeding informatics].

Chu et al. highlight a comparison of different marker systems in bread wheat and the influence on genetic diversity and the prediction ability. While array-based SNP markers showed an ascertainment bias leading to underestimation of diversity within the population, GBS derived markers showed the highest potential as the method of choice for (pre-)breeding programs [categories (1) Biodiversity and information systems and (2) Breeding informatics].

Grehl et al. showed the strengths and weaknesses of different mapping tools for whole-genome bisulfite sequencing in several plant species in both simulated data sets (*Arabidopsis thaliana, Brassica napus, Glycine max, Solanum tuberosum*, and *Zea mays*) as well as the real-world data of *Glycine max*. They recommend using BSMAP (Xi and Li, 2009) for its speed and high precision and Bismark (Krueger and Andrews, 2011) for its memory footprint, high precision and the high number of uniquely mapped reads [category (1) Distributed computing, tools, and infrastructures].

Anderson and Murray developed an open-source R function (R/UAS::plotshpcreate) to enable the detection of small plots with remote sensing technologies, such as Unoccupied Aerial Systems (UAS). This allows the creation of multi-polygon shapefiles that also contain information about the experimental design, field orientation and plot dimensions [categories (1) Distributed computing, tools and infrastructures, and (2) Image-based analysis and data visualization].

Lee et al. presented a new genome sequence assembly for winter oilseed rape (*Brassica napus*) accession “Express 617.” They used a complex sequencing and assembly strategy with a backbone of 50x Pacific Biosciences long reads, supported by Illumina short-reads, optical map data and genetic maps [relating to categories (1) Biodiversity and information systems and (2) Breeding informatics].

In their article, Santantonio et al. articulate and analyze the potential and challenges of implementing Genomic Selection (GS) in the public breeding programs of developing countries. Proof-of-concept studies were conducted by ICRISAT and CIMMYT in chickpea and maize to examine potential approaches for GS implementation. The authors also discuss the need to develop breeding informatics capabilities to realize large-scale genomic breeding strategies. As an outcome, the authors recommend a multi-phased implementation of GS, (1) building informatics capabilities, optimizing trail design to build cost-effective training sets, (2) increasing selection intensity in the early stage variety development pipeline, early recycling of lines as parents and reducing the number of testing seasons before variety release, and (3) implementation of rapid-cycle recurrent selection to reduce generation intervals toward the biological limits of the species. In this stepwise approach, the genotyping of lines will deliver a series of benefits from the very beginning of the implementation [categories (1) Biodiversity and information systems and (2) Breeding informatics].

Holtgräwe et al. presented a new draft genome assembly of the vitis rootstock “Börner,” which is of particular interest for breeders, as this hybrid carries several resistance loci against downy mildew. Using a combination of different short-read sequencing technologies (454 and Illumina) and the incorporation of BAC end sequences, they succeeded in partially separating the resulting contigs into two haplophases. In addition, they generated molecular markers (SNVs and SSRs) and were able to use this new resource to narrow down the position of the resistance locus *Rpv14* (Ochssner et al., 2016) to <0.5 Mbp on chromosome 5 [categories (1) Biodiversity and information systems and (2) Breeding informatics].

In their study, Narisetti et al. presented an algorithm for segmentation/detection of wheat spikes that relies on a pre-trained neural network classifier. Previous similar approaches (Qiongyan et al., 2017) applied to images of European wheat cultivars failed to detect spikes growing in the middle of the plant surrounded by multiple leaves of similar color, and textural features. To enhance detection of spikes by suppressing linear leaf contours the Frangi edge filter (Frangi et al., 1998) was applied [categories (1) Distributed computing, tools and infrastructures and (2) Image-based analysis and data visualization].

König et al. implemented a sophisticated web-based visual analysis tool that enabled them to impressively illustrate the high diversity of plant genetic resources of barley species contained in genebanks around the world [categories (1) Biodiversity and information systems, (2) Distributed computing, tools and infrastructures, (3) Breeding informatics and (4) Image-based analysis and data visualization].

## Conclusions

The symbiotic relationship between bioinformatics and plant sciences not only leads to a better understanding of crop biology, it also enriches bioinformatics with powerful methods, theoretical approaches, standards, and software tools. While this RT on Applied Bioinformatics in Crops only covers a few of the recent developments, the overall progress that has been made in recent years is enormous and manifold. These developments range from advances in distributed computing, tools and infrastructures as a backbone for all bioinformatics work, to new or updated information systems mainly in the frame of biodiversity studies. There has been progress in using breeding informatics to facilitate advances in genome sequence analysis, and new methods and tools for image-based analysis and data visualization have proved indispensable for high-throughput phenotyping. The high degree of interdisciplinarity, which becomes apparent when looking at the categories and topics of the contributions for this RT, is worth mentioning and can be seen as evidence of good connectivity within this community. All advances mentioned are crucial to accelerating the development of stress-tolerant elite crops and to improving breeding strategies, both for increased yield and yield stability, as well as rapid breeding cycles. This progress will also depend on the availability of multi-omic and phenomic data sets, particularly training data, to guarantee precise predictions, and the adoption of new bioinformatics technologies. The RT does not cover the topic of systems biology and modeling in crop and plant sciences, which is essential for the progress of the entire research field as we learned during several discussions among conference participants. It is crucial to link the observed genomic and phenotypic variation and to integrate multimodal omic data into coherent frameworks to unravel and understand underlying molecular processes. These integrative models allow for a systems-level understanding and enable research to easily test hypotheses, as well as the effects of the disturbances and perturbations directing field and wet-lab experiments. They pave the way for the development of elite crops and will help breeders to intelligently and rapidly adapt breeding strategies.

## Author Contributions

M-AB, SB, JS, EG, and US co-wrote this editorial based on the contributions to this Research Topic. All authors contributed to the article and approved the submitted version.

## Conflict of Interest

The authors declare that the research was conducted in the absence of any commercial or financial relationships that could be construed as a potential conflict of interest.
